# High-Pressure Homogenization for Enhanced Bioactive Recovery from Tomato Processing By-Products and Improved Lycopene Bioaccessibility during In Vitro Digestion

**DOI:** 10.3390/antiox12101855

**Published:** 2023-10-13

**Authors:** Serena Carpentieri, Giovanna Ferrari, Francesco Donsì

**Affiliations:** 1 Department of Industrial Engineering, University of Salerno, Via Giovanni Paolo II, 132, 84084 Fisciano, Italy; scarpentieri@unisa.it (S.C.); gferrari@unisa.it (G.F.); 2 ProdAl Scarl, c/o University of Salerno, Via Giovanni Paolo II, 132, 84084 Fisciano, Italy; 3 NBFC (National Biodiversity Future Center), 90133 Palermo, Italy

**Keywords:** High-Pressure Homogenization, tomato residues, zero-waste economy, techno-functional properties, bioactive compounds, lycopene bioaccessibility

## Abstract

The principles of industrial ecology have emerged as pivotal drivers of eco-innovation, aiming to realize a “zero-waste” society where waste materials are repurposed as valuable resources. In this context, High-Pressure Homogenization (HPH) presents a promising, easily scalable micronization technology, capable of enhancing the extractability and bioaccessibility of bioactive compounds found in tomato processing by-products, which are notably abundant waste streams in the Mediterranean region. This study focuses on optimizing HPH treatment parameters to intensify the recovery of bioactive compounds from tomato pomace. Additionally, it investigates the multifaceted impacts of HPH on various aspects, including color, particle size distribution, microscopic characteristics, surface properties, bioactivity, and lycopene bioaccessibility through in vitro digestion simulations. The results demonstrate that the application of HPH under optimized conditions (80 MPa, 25 °C, 10 passes) induces a remarkable 8-fold reduction in mean particle size, reduced surface tension, improved physical stability, uniform color, increased total phenolic content (+31%), antioxidant activity (+30%), dietary fiber content (+9%), and lycopene bioaccessibility during the intestinal digestion phase compared to untreated samples. These encouraging outcomes support the proposition of integrating HPH technology into an environmentally friendly industrial process for the full valorization of tomato processing residues. By utilizing water as the sole solvent, this approach aims to yield a functional ingredient characterized by greater nutritional and health-promoting values.

## 1. Introduction

The residues generated by the agri-food industry often present significant environmental challenges in terms of their impact and disposal [1]. In Europe, the principles of industrial ecology have emerged as guiding principles for eco-innovation, with the ultimate goal of realizing a “zero-waste” society in which these residues are repurposed as valuable raw materials for new products and applications [2]. This approach is exemplified by the concept of industrial symbiosis, where the aim is to utilize waste materials from one sector as inputs for other sectors [3]. In this context, the cultivation of tomatoes, a widely cultivated vegetable crop in Mediterranean countries, plays a crucial role [4]. The industrial processing of tomatoes into products like tomato juice, paste, puree, and ketchup [5] generates substantial quantities of by-products, including tomato peels, seeds, and unused pulp, collectively constituting 2–5% of the total processed tomatoes by weight [6]. For example, in the Campania region (Southern Italy) alone, an estimated 44,400 tons of tomato peels (equivalent to about 9000 tons of dry peels) are produced annually, containing about 6.5 tons of lycopene [7].

These tomato by-products have attracted attention in numerous studies due to their high nutritional value, characterized by significant amounts of vitamins, proteins, phenolic compounds, dietary fibers, and carotenoids. On a dry basis, the tomato processing by-products (peels and seeds) are rich in polyphenols (>1000 mg GAE/kg [8]), dietary fibers (between about 50 wt% [8] and 89 wt% [9]), proteins (between 18 wt% [8] and 40 wt% [9]), oil content (about 21 wt% [9]) and carotenoids, particularly lycopene (about 500–800 mg/kg), accounting for 80–90% of the total carotenoids (in a 1:5 ratio, *w*/*w*, in the seeds and skins, respectively) [10].

Notably, lycopene has gained prominence for its potential health benefits, such as its role as a potent free radical scavenger and its demonstrated antioxidant, antimutagenic, hypolipidemic, and anticarcinogenic properties, as shown by in vitro and in vivo studies [11]. Due to its properties, lycopene consumption has been linked to a reduced risk of prostate, pancreatic, and stomach cancer [9]. The high antioxidant activity of lycopene, resulting from its polyene structure with 11 conjugated double bonds, is the main reason for these health-related effects [12].

Lycopene is situated within vesicles formed by the inner membrane of the plastid, and it is found in the inner portion of the lipid bilayer, either in the form of carotenoid-protein complexes or as solid microcrystals [13]. Therefore, extracting lycopene from tomato peels traditionally involves intensive thermal or mechanical treatments and the use of organic solvents, which can have adverse environmental implications [6]. To address this challenge, green technologies such as High-Pressure Homogenization (HPH) have been explored within the food processing industry. HPH has proven effective in micronizing plant tissues in suspension, thereby facilitating the extraction of bioactive compounds trapped in cells, and altering the properties of macromolecules and their size distribution in foods [6], due to the combination of fluid mechanical effects such as cavitation, turbulence, shear and elongational stresses [14,15].

In the context of tomato product processing, HPH has been extensively used to produce nanosuspensions with enhanced solubility, stability, and bioavailability [6,13,15,16,17,18,19]. This approach has yielded several advantages, including increased viscosity, improved physical stability, and uniform texture and color [13,15,18]. However, the application of carotenoids in foods, which have the potential to reduce the incidence of certain chronic diseases, is currently limited. This limitation stems from their poor water solubility, susceptibility to chemical degradation, and low bioavailability, all of which significantly impact their health-promoting effects [13].

Recent studies have explored the impact of HPH on carotenoid bioaccessibility and stability in various matrices, revealing somewhat contradictory findings. On one hand, reducing particle size through tissue disruption has been demonstrated to enhance carotenoid bioaccessibility [19,20]. On the other hand, the increased consistency resulting from polymer–polymer interactions that form a fiber network can potentially entrap carotenoids, thereby reducing their bioaccessibility [13,16,17]. Additionally, research has indicated that increasing homogenization pressure can disrupt tomato cell aggregate structures while simultaneously strengthening the fiber network. This dual effect has been shown to lead to a decrease in lycopene bioaccessibility, as shown for tomato juice [19], and tomato pulp [5,13,16].

However, the majority of studies have primarily focused on the effects of HPH on lycopene bioaccessibility, rheology, and physical properties in whole tomatoes, tomato pulp, puree, juice, and ketchup, with limited attention given to tomato processing by-products.

This study aims to bridge this gap by investigating the effects of HPH on tomato by-products, aligning with the “zero-waste” concept and their potential reuse within the food industry. Its primary objective is to explore the complete recovery and valorization of cost-effective sources of high-value compounds, such as tomato by-products, while adhering to the “zero-waste” policy. This is achieved through the utilization of HPH as a green and sustainable technology, employing water as a solvent. Furthermore, this study seeks to determine the optimal processing conditions for enhancing the recovery of intracellular compounds, including phenolic compounds and lycopene, and elucidating the impact of HPH-induced structural changes on the physical properties of processed suspensions. Additionally, the in vitro bioaccessibility of lycopene, with a view towards its potential exploitation and application, is assessed.

## 2. Materials and Methods

### 2.1. Raw Materials and Chemicals

Fresh tomato pomace was supplied by a local canning company (Salvati Mario & C. S.p.a.—Fontanella, Mercato San Severino, Italy). The samples were collected during the summer campaign of 2022, transported to the laboratories of ProdAl Scarl (Fisciano, Italy), and preserved by freezing at −20 °C until required. All solvents, reagents, and standards utilized in the analyses were procured from Sigma Aldrich (Steinheim, Germany).

### 2.2. Proximate Composition

The moisture content was determined using the gravimetric method in an oven (Heraeus Group, Hanau, Germany) set at 105 °C until a constant mass was reached, following the AOAC 2003 standard procedure. Protein, ash, fat, and total dietary fiber contents were determined in accordance with the AOAC official methods 920.152, 923.03, 922.06, and 991.43, respectively. Total carbohydrates were evaluated by deducting the sum of the percentages of the other proximate components from a 100 g sample. All the results were reported on a dry weight basis.

Additionally, a preliminary analysis was conducted to detect the presence of pesticides, using the Agri-Screen Ticket Pesticide Detection Kit (Neogen corporation, Lesher Place, Lansing, MI, USA), a method approved by AOAC International, AOAC Research Institute, AFNOR Certified, IUPAC, USDA/GIPSA (FGIS) USDA/FSIS.

### 2.3. Sample Preparation or Pre-Processing

The fresh tomato pomace was milled using a laboratory knife grinder and then suspended in pure water at a solid-to-liquid ratio of 1:10 g/mL. This suspension was subsequently subjected to high-shear mixing (HSM) at 20,000 rpm for a duration of 10 min. The HSM was carried out using a T–25 Ultra Turrax device (IKA^®^–Werke GmbH & Co. KG, Staufen, Germany) equipped with an S25–N18 G rotor, all while maintaining a constant temperature by immersing the device in an ice bath. The sample pretreated with HSM was used as the control, representing np = 0 (0 HPH passes), in order to evaluate the impact of the HPH treatment.

### 2.4. HPH Treatment: Experimental Set-Up

The obtained suspension underwent HPH treatment using a laboratory-scale homogenizer, developed in-house [6], equipped with an orifice valve (model WS1973, Maxi-mator JET GmbH, Schweinfurt, Germany) featuring interchangeable orifices within the size range 80–100 μm to regulate the pressure drop. An air-driven Haskel pump (model DXHF–683, EGAR S.r.l., Milan, Italy) was employed as a pressure intensifier. The suspension was subjected to High-Pressure Homogenization (HPH) processing under varying conditions, including treatment temperature (ranging from 5 to 50 °C), pressure (P = 80–100 MPa), and the number of passes through the orifice (np = 1–15). To maintain temperature stability and prevent any temperature rise caused by frictional heating, the dispersions were cooled to the desired temperatures using two tube-in-tube exchangers. One exchanger was positioned between the pump and the homogenization valve, and the other was placed immediately downstream of the valve. Additionally, the feeding tank was equipped with a cooling jacket set to the target temperature for further temperature control.

To determine the optimal HPH processing conditions that maximize the recovery of total phenolic content (TPC), antioxidant activity as ferric reducing activity (FRAP), and lycopene content in tomato pomace suspensions, both untreated (control) and HPH-treated samples, the response surface methodology (RSM) was employed. The experimental design encompassed 24 runs, as detailed in Table 1. A two-factor interaction (2FI) model was employed, as represented in Equation (1), to predict the response variables as a function of the investigated independent variables, using Design Expert 12 (StatEase, Minneapolis, MN, USA).

(1) $$Y_{k}=\alpha_{0}+{\sum_{i=1}^{3}\alpha_{i}X}_{i}+\sum_{i=1}^{3}\sum_{j=i+1}^{4}\alpha_{ij}X_{i}X_{j}+\sum_{i=1}^{3}\sum_{j=i+2}^{5}\alpha_{ij}X_{i}X_{j}$$

where Y_k_ represents the predicted response variable, X_i_ and X_j_ represent the independent factors, and α_0_, α_i_, and α_ij_ are the intercept, and regression coefficients of the linear, and interaction terms of the model, respectively.

After the HPH treatment was completed, the suspension underwent a concentration process using an R–200/205 Rotavapor (BÜCHI Labortechnik AG, Flawil, Switzerland) until it achieved a volume reduction of up to 80%. Subsequently, the concentrated suspension was subjected to freeze-drying in a 25 L VirTis Genesis freeze-drier (SP Scientific Products, Stone Ridge, NY, USA). The resulting HPH freeze-dried extract was subsequently packed in vacuum-sealed flexible bags and stored under refrigerated conditions for further analyses.

### 2.5. Analyses of the Obtained Suspensions

#### 2.5.1. Total Phenolic Content (TPC)

The TPC of the extracts was determined using the Folin–Ciocalteau assay, following the procedure outlined in a recent study by Carpentieri et al. (2023) [21]. The TPC values were expressed as milligrams of gallic acid equivalent per gram of sample dry weight (mgGAE/g _DW_).

#### 2.5.2. Ferric Reducing Antioxidant Power (FRAP)

To assess the FRAP of the extracts, the procedure detailed in the study by Carpentieri et al. (2023) was followed [21] was followed. FRAP values were quantified and expressed as milligrams of ascorbic acid equivalent per gram of the sample’s dry weight (mgAAE/g _DW_).

#### 2.5.3. Lycopene Determination

The lycopene content of the samples was determined using a modified version of the reduced volume lycopene assay originally described by Fish et al. in 2002 [22]. In this modified method, a mixture comprising 0.05% (*w*/*v*) BHT in acetone, 95% ethanol, and hexane, in a ratio of 1:1:2, was added to the sample at a solid-to-liquid ratio of 0.05 (g/mL). Subsequently, the vials were covered and placed on an orbital shaker at 180 rpm for 15 min. Following this, 3 mL of distilled water were added to each vial, and the samples were shaken for an additional 5 min. Shaking was then discontinued, allowing the vials to stand at room temperature for 5 min to facilitate phase separation. The absorbance of the upper hexane layer was measured at 503 nm, and the lycopene content was calculated and expressed as milligrams of lycopene per gram of the sample’s dry weight (mg/g _DW_).

### 2.6. Colorimetric Parameters

The absolute measurements of lightness (L*), redness (a*), and yellowness (b*) coordinates of the suspensions were determined using the CR–400 colorimeter (Konica Minolta Inc., Tokyo, Japan), following the official CieLab method.

To quantify the color difference (ΔΕ*) between the HPH-treated and untreated samples, Equation (2) was employed. Additionally, the chroma (C*_ab_) and the hue angle (h*_ab_) were evaluated according to Equation (3) and Equation (4), respectively.

(2) $${\Delta E}^{*}=\sqrt{{\left(\Delta L^{*}\right)}^{2}+({\Delta a^{*})}^{2}+({\Delta b^{*})}^{2}}$$

(3) $${C^{*}}_{ab}=\sqrt{a^{*2}+b^{*2}}$$

(4) $${h^{*}}_{ab}=\arctan\left(\frac{b^{*}}{a^{*}}\right)$$

### 2.7. Particle Size Distribution (PSD)

The PSD of untreated and HPH-treated suspensions was analyzed by laser diffraction using a MasterSizer 2000 particle size analyzer (Malvern Panalytical, Malvern, UK). Water at 25 °C, with a refractive index of 1.33, was used as the dispersant medium.

Characteristic diameters, including d(0.1), d(0.5), and d(0.9), corresponding to the 10th, 50th, and 90th percentiles of the cumulative size distribution of the suspensions, were determined. Additionally, the mean particle size, expressed as the volume moment mean diameter D[4,3], and the surface moment mean diameter D[3,2], were evaluated.

### 2.8. Optical Microscopy Analysis

The effectiveness of the HPH treatment in disrupting plant cells was examined by conducting microscopic analysis on both the untreated and HPH-treated suspensions. Microscopic examination was performed using an optical inverted microscope (Nikon Eclipse TE 2000S, Nikon Instruments Europe B.V., Amsterdam, The Netherlands). The microscope was equipped with a polarization filter and utilized a 10× objective lens, coupled to a DS Camera Control Unit (DS–5M–L1, Nikon Instruments Europe B.V.), for image acquisition.

### 2.9. Interfacial Tension

The interfacial tensions of supernatants recovered from both the untreated and HPH-treated suspensions. The supernatants were obtained through centrifugation at 5 °C and 6500 rpm (5300× *g*) for 10 min, followed by filtration using Whatman no. 4 filter paper. The measurements were carried out using the pendant drop method with the assistance of a contact-angle meter (LTD CAM 200, KSV Instruments, Helsinki, Finland), equipped with an image software analyzer (CAM 101, KSV Instruments).

In this method, a syringe fitted with a stainless-steel needle (0.71 mm in diameter) was filled with the suspensions and immersed into the oil phase. The optical contact angle meter recorded changes at the water/oil interface, and measurements of the interfacial tension (σ) were performed over a duration of 2000 s.

### 2.10. Thermal Stability Kinetics

The freeze-dried extracts were then subjected to thermal stability analysis, which included assessments of total polyphenols (Section 2.5.1), antioxidant activity (Section 2.5.2), and lycopene content (Section 2.5.3). For analysis preparation, the extracts were dissolved in distilled water and positioned in an oven (Heraeus, Hanau, Germany). These samples were exposed to four distinct temperatures (25 °C, 40 °C, 70 °C, 100 °C).

At various time intervals ranging from 60 min to 8 h, samples were withdrawn from the extracts for subsequent analysis, aimed at establishing thermal stability kinetics. The resulting data were expressed over time per gram of the dry extract as milligrams of gallic acid equivalent (mgGAE/g _DW_), milligrams of ascorbic acid equivalent (mgAAE/g _DW_), and milligrams of lycopene (mg/g _DW_), corresponding to TPC, antioxidant activity, and lycopene content, respectively.

### 2.11. Simulated Gastrointestinal Digestion

The evaluation of simulated gastrointestinal digestion was carried out on both untreated and HPH-treated suspensions. This assessment followed the international consensus procedure, with minor modifications as outlined by Carpentieri et al. (2023) [21].

#### Lycopene Release

Samples were collected at specific time points during the simulated gastrointestinal digestion, including after the oral phase, at 60 and 120 min of the gastric phase, and 20 and 120 min of the intestinal phase. These collected samples were subsequently subjected to centrifugation at 14,000 rpm (20,800× *g*) at 4 °C for 20 min using an Eppendorf centrifuge (Micro Centrifuge 5417R, Eppendorf Srl, Milan, Italy).

The determination of lycopene content followed the method described in Section 2.5.3. To calculate lycopene release from the samples, Equation (5) was employed:

(5) $${Release}_{Lycopene}=\frac{{Lycopene}_{i}}{{Lycopene}_{T}}\times 100$$

where *Lycopene_i_* is the lycopene content detected in each sample during the digestive phases, and *Lycopene_T_* is the lycopene content measured in the undigested sample.

### 2.12. Statistical Analysis

All experiments and analyses were conducted in triplicate, and the results are presented as means ± standard deviations. To assess differences among the mean values, a one-way analysis of variance (ANOVA) was performed using the SPSS 20 statistical package (SPSS IBM, Chicago, IL, USA). Post–hoc Tukey tests were conducted to identify statistically significant differences (*p* < 0.05).

## 3. Results and Discussion

### 3.1. Proximate Composition of Tomato Pomace

The results of the proximate analysis for the investigated raw material, tomato pomace, are presented in Table 2.

Despite the inherent variability associated with plant materials due to factors such as crop variety, ripeness, climate, soil, and water management systems, as well as pre- and post-harvest agricultural practices, it is noteworthy that the ash, protein, fat, carbohydrate, and total dietary fiber contents detected in this study are consistent with the ranges reported in the literature for tomato pomace [23,24,25].

Furthermore, a significant challenge in the development of natural extracts from agrifood residues pertains to the uncertainty surrounding the quality of secondary raw materials, especially concerning the presence of chemical substances that may pose risks to human health.

Hence, the preliminary analysis conducted to detect the presence of pesticides, utilizing an official method based on a straightforward biochemical principle, confirmed that the analyzed sample was devoid of pesticides or contained quantities well below the established maximum residue levels (MRL) for human consumption, as stipulated by Regulation (EC) No. 396/2005 (clear blue response in Table 2).

### 3.2. Effect of HPH Processing Conditions on the Unlocking of Bioactive Compounds from Tomato Pomace

#### 3.2.1. Model Fitting

In this study, the experimental design outlined in Table 1 was employed to examine the impact of three independent variables: processing temperature, pressure, and the number of passes through the homogenization valve, on the total phenolic content (TPC), antioxidant activity (FRAP), and lycopene content of aqueous suspensions of tomato pomace subjected to HPH treatment. To facilitate comparison, untreated tomato pomace suspensions (control) were also included in the analysis.

The results obtained reveal that all three independent factors significantly influenced the measured response variables. To quantify the influence of these factors on the release of TPC and lycopene from tomato pomace with high antioxidant activity, a two-factor interaction (2FI) model (Equation (1)) was employed to fit the experimental data derived from the design for all the response variables under investigation (see Table 1). Table 3 provides the values and significance of the regression coefficients, *p*-values, the determination coefficient (R^2^), and the root mean square error (RSME) for each variable.

The results reveal that all the examined factors had a statistically significant linear effect on the TPC, FRAP values, and lycopene content of the analyzed suspensions.

In particular, TPC was found to be less influenced by processing temperature compared to antioxidant activity and lycopene content. Conversely, temperature and the number of passes had a more pronounced impact on lycopene content than on the other factors investigated.

It is worth noting that, concerning the interactions between individual factors, no significant effects were observed for TPC and FRAP values. However, in the case of lycopene content, all interactions were slightly significant, indicating dependencies between processing temperature and pressure, as well as between pressure and the number of passes.

The ANOVA presented in Table 3 indicates that the root mean square error (RSME) values were consistently lower than 0.041, while the determination coefficient (R^2^) values ranged from 0.703 to 0.843. These findings support the predictive reliability of the selected model, which was significant (*p* ≤ 0.0009) for all the investigated responses.

To visually validate the model, parity plots were generated (see Figure S1 in the Supplementary Material), displaying the actual (experimental) values of (a) TPC, (b) antioxidant activity, and (c) lycopene content, in comparison to the predicted values obtained using the response surface method. These plots further affirm the strong correlation between the predicted and experimental results.

#### 3.2.2. Optimization of the HPH Processing Conditions

**Figure 1:**
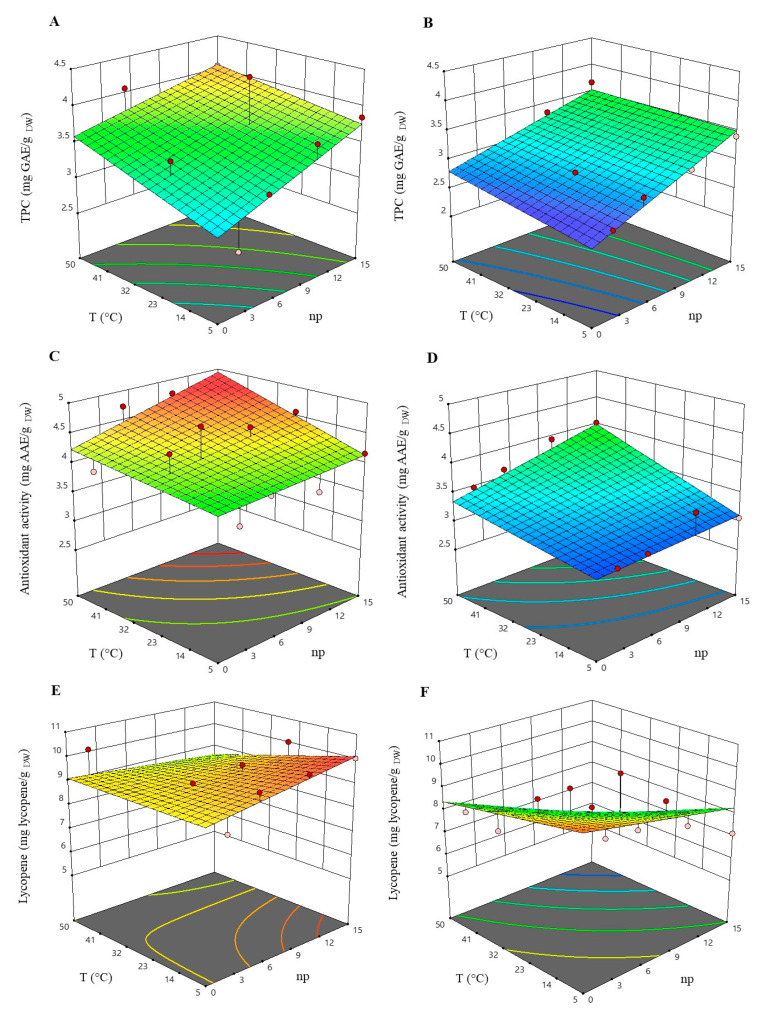
Response surfaces for TPC, antioxidant activity, and lycopene content in the untreated and HPH-treated suspensions at 80 MPa (**A**,**C**,**E**) and 100 MPa (**B**,**D**,**F**) as a function of the process temperature and the number of passes through the homogenization valve.

The impact of temperature, pressure, and the number of passes through the valve on the levels of TPC, antioxidant activity, and lycopene content is visually presented in the Response Surface Plots shown in Figure 1.

In general, the highest levels of all response variables were achieved at the lowest applied pressure (80 MPa), after 10 passes through the valve, and at an intermediate temperature of 25 °C. The results also demonstrate that HPH treatment under these optimal conditions significantly increased the extractability of TPC (+31%), FRAP values (+25%), and lycopene content (+42%) compared to the control (untreated suspension). This effectiveness of HPH treatment in enhancing lycopene release from tomato pomace suspensions was further confirmed by the HPLC–PDA chromatogram displayed in Figure S2 (Supplementary Material), which showed a +45% increase in the lycopene peak compared to the control.

Similar findings regarding the performance of HPH in unlocking bioactive intracellular compounds from tomato peels were reported by Juric et al. (2019) [6]. They observed an increased release of polyphenols (+32.2%) and antioxidant activity (+23.3%) in tomato peel aqueous suspensions treated with HPH (10 passes) compared to the control sample. HPH treatment also led to the enhanced release of water-insoluble lycopene into the aqueous supernatant [6].

In the explored parameter range, the TPC level exhibited a linear increase with rising temperature and the number of passes through the valve (+34% compared to the untreated suspension). A similar trend was observed for the level of antioxidant activity (+28% compared to the untreated suspension). These findings align with those reported by several authors who observed a linear dependence of temperature and processing time on TPC and antioxidant activity levels [26].

Higher temperatures generally enhance the solubility and diffusivity of intracellular phenolic compounds in the solvent. However, it is worth noting that subjecting plant materials to temperatures exceeding 50 °C may induce thermal oxidation or degradation of certain phenolic compounds [27].

Regarding lycopene content, it appeared to be most affected by the independent variables. Its concentration decreased with increasing temperature up to 50 °C and pressure up to 100 MPa (−17% on average compared to the untreated suspension). The most significant reduction in lycopene content was observed when combining the highest pressure, temperature, and number of passes investigated (100 MPa, 50 °C, and 15 passes, respectively). Several studies have concluded that temperature plays a significant role in lycopene concentration during processing or drying, and excessive heat treatment can easily lead to its reduction [28,29]. High temperatures can lead to the release of hydroxyl enzymes that may degrade lycopene, though lycopene is less susceptible to degradation under milder heat exposure. However, in combination with pressure and treatment time, it can contribute to the reduction in lycopene content [28].

The reduction in particle size, resulting from the disruption of plant tissue caused by fluid-mechanical stresses induced by HPH treatment, has been shown to enhance carotenoid recovery and bioaccessibility [6,19,30]. Conversely, the increase in consistency, attributed to the formation of a fiber network through polymer–polymer interactions, has been demonstrated to trap carotenoids and reduce their extraction [13]. Consistent with the findings of this study, a decreasing trend in lycopene recovery and bioaccessibility with increasing homogenization pressure was observed, which could be attributed to the breakdown of cell aggregate structures, making lycopene more susceptible to degradation [13].

It is important to note that the lycopene values (ranging from 5.81 to 10.20 mg/g _DW_) obtained in this study align with those reported by other researchers, albeit with slight variations, depending on factors such as tomato variety, ripeness, and experimental protocols [6,30,31].

### 3.3. Effect of HPH Processing Conditions on the Color of Tomato Pomace Aqueous Suspensions

Color is a crucial sensory attribute that indirectly reflects various quality aspects of food, including flavor and pigment content. It significantly impacts consumers’ perception of food quality [32]. Therefore, the color parameters of tomato pomace suspensions under different HPH processing conditions (P = 80 MPa, T = 5–50 °C, np = 0–15) were systematically examined and are presented in Table 4.

The results revealed that all analyzed samples exhibited positive values of a* (indicating redness), falling within the range of 11.69 to 14.86. This indicates that the dominant color component in all cases was red [33]. However, when the treatment temperature was increased to 50 °C, and after 15 min of processing, the a* values of HPH-treated suspensions significantly decreased (*p* < 0.05) by approximately 40% compared to the untreated sample. Conversely, and in line with the detected lycopene content (Table 1), a* values increased when treating the suspensions at 25 °C, with increasing treatment time (+20% compared to the control).

The b* parameter, which accounts for the tendency toward yellow, exhibited a slight increase with processing time, of 17%, 10%, and 23% at 5 °C, 25 °C, and 50 °C, respectively, compared to the untreated sample. These observations indicate the influence of processing conditions on the potential discoloration of the suspensions, particularly at higher temperatures. In this context, Kaur et al. (2006) also reported a direct relationship between visual color and lycopene content [34].

To assess whether the changes in color parameters that were induced by the treatment conditions were perceptible to the human eye, the ΔE* parameter was evaluated by comparing each HPH-treated suspension with the untreated one. Consistent with the previously discussed color parameters, ΔE* values ranged between 1.93 and 7.72, significantly increasing by about 50% under more severe processing conditions at 50 °C, after 15 passes through the valve (Table 4). While the color change in HPH-treated suspensions is noticeable to the human eye [33], the lowest ΔE* values were observed for samples treated at 25 °C (ΔE* = 1.93–4.36).

Chroma (C*_ab_) and hue angle (h*_ab_) are quantitative and qualitative color attributes, respectively. Chroma provides information about color vividness, while the hue angle defines colors in terms of traditional categories such as reddish, orange, and yellowish (e.g., 0° for red, 90° for yellow, 180° for green, and 270° for blue), as perceived by the human eye [32]. Specifically, treatment temperature did not induce significant changes in chroma; however, the hue angle, which ranged from 56.21° to 70.13° (within the red-yellow region), slightly increased (+21%) as treatment time and temperature increased, shifting towards higher yellowness. Conversely, hue angle values decreased slightly when treating the suspension at 25 °C and 80 MPa, shifting towards the red component. This observation aligns with the enhanced release of lycopene following HPH treatment.

These results are consistent with those reported by Kubo et al. (2013) [33] and Liu et al. (2019) [35] who observed that HPH-treated tomato juice and lily pulp appeared brighter, clearer, more saturated in red and yellow, exhibited more intense color, and displayed increased ΔE values upon HPH treatment as pressure and treatment time increased.

### 3.4. Effect of HPH Processing Conditions on the Particle Size of Tomato Pomace Aqueous Suspensions

**Figure 2:**
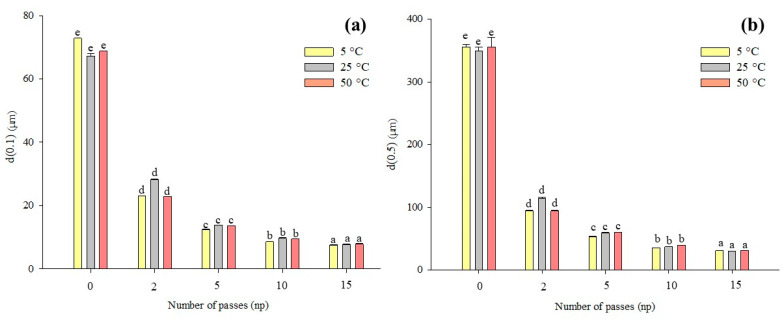

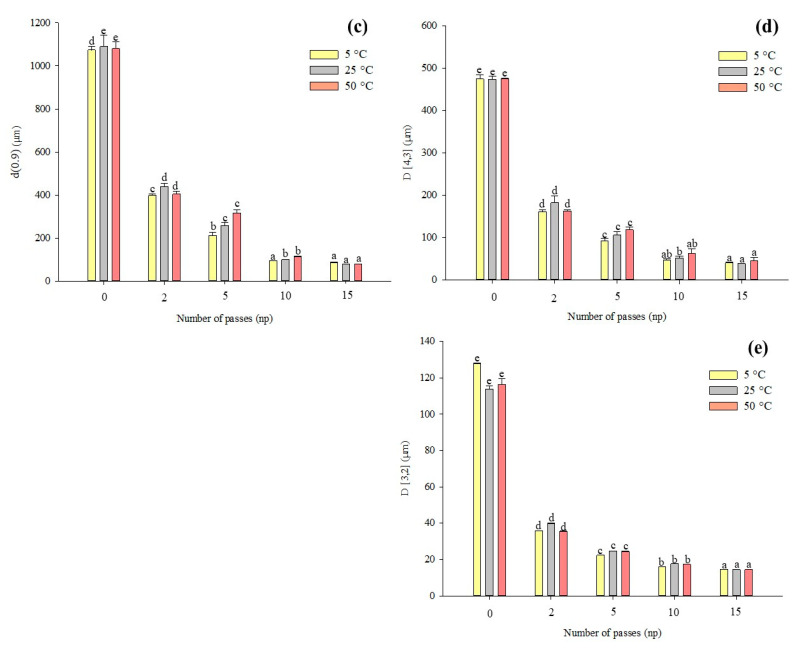
Characteristic diameters (μm) (d(0.1), (**a**); d(0.5), (**b**); d(0.9), (**c**); D[4,3], (**d**); D[3,2], (**e**)) of untreated (np = 0), and HPH-treated (P = 80 MPa, T = 5–50 °C, np = 2–15) tomato pomace aqueous suspensions. Different letters above the bars of the suspension at the same temperature indicate significant differences among the mean values of the samples (*p* ≤ 0.05).

The particle size characteristics of the tomato peel suspension are presented in Figure 2, depicting the values of d(0.1), d(0.5), d(0.9), D[4,3], and D[3,2] concerning treatment intensity and processing temperature, with a fixed pressure of 80 MPa. As anticipated, HPH treatment resulted in a reduction in characteristic diameters, consistent with observations in various plant matrices, including tomato juice [33,36], peels [6], puree [18], taro pulp [37], mango juice [38], and lily pulp [35].

**Figure 3:**
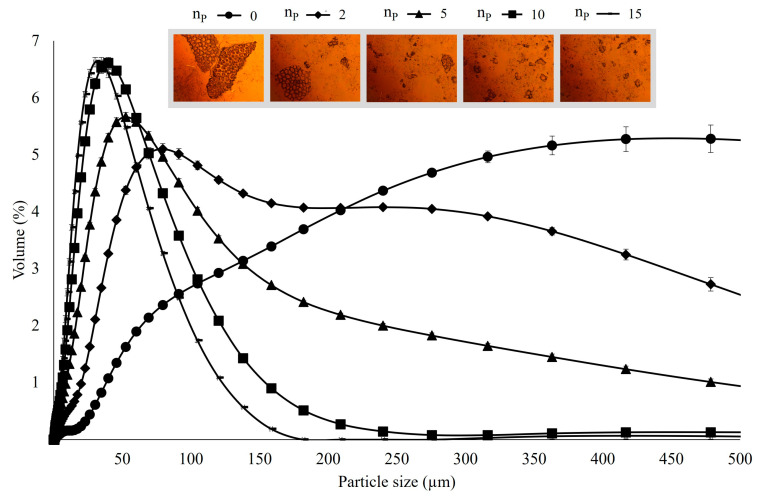
Particle size distribution (PSD) and micrographs at 10× of untreated (Control, np = 0), and HPH-treated (P = 80 MPa, T = 25 °C, np = 2–15) suspensions.

Specifically, it is evident that the diameters d(0.1), d(0.5), and d(0.9), corresponding to the 10th, 50th, and 90th percentile of the cumulative distribution, respectively, progressively decreased. This led to narrower size distributions of tomato pomace suspensions with an increasing number of passes through the HPH valve (Figure 3). A remarkable reduction in d(0.1) was observed, dropping from 70 to 9 µm on average for HSM and 10 HPH passes, respectively. Similarly, there was a progressive reduction in the median value, d(0.5), and d(0.9) of the distribution, with values decreasing from 354 to 38 µm and from 1081 to 103 µm, respectively. These findings corroborate those reported by Juric et al. (2019) [6], who noted a decrease in d(0.1) from 331 to 31 µm and in d(0.9) from 1007 to 111 µm for HSM and 10 HPH passes tomato peel suspensions.

Comparable results were also obtained by Tan and Kerr (2015) [18], and Kubo et al. (2013) [33] for tomato puree and juice, respectively. They observed that increasing the number of passes resulted in further reductions in particle size. However, the majority of the size reduction occurred after the second pass, with only modest changes occurring after 5 passes. Therefore, a higher number of passes had a diminishing effect on particle size [38]. The homogenization process disrupts the remaining cells and fragments their components into small, suspended particles. Smaller fragments are expected to be less susceptible to further processing than larger ones [6,33], a point that is visually evident in the microscopic images presented in Figure 3.

When considering the size distribution in terms of volumetric-weighted (D[4,3]) and surface-weighted (D[3,2]) mean diameters, the results show that HPH treatment induced a significant reduction in the mean particle size of the suspensions. A drastic reduction in D[4,3] was observed, dropping from 474 to 172 µm after just 2 passes through the valve, and achieving an 8-fold reduction (51 µm) after 10 passes due to cell fragmentation. In line with the significant decrease in D[4,3] compared to control samples, the particle size distribution exhibited a mono-modal curve in HPH-treated suspensions (Figure 3), indicating a pronounced shift toward smaller sizes compared to untreated cells. Furthermore, both D[4,3] and D[3,2] showed a decreasing trend in mean particle size and a significant increase in sample homogeneity as treatment time increased.

Regarding the effect of temperature on particle size distribution, it is noteworthy that at 50 °C, D[4,3] exhibited a slight increase after 5 passes. This can be attributed to the potential coagulation of proteins induced by higher processing temperatures, as previously reported [38]. However, although both D[4,3] and D[3,2] were reduced by HPH processing, D[4,3] showed progressive reduction as the number of passes increased, whereas D[3,2], after 5 passes, exhibited only a slight, albeit statistically significant, change. This distinct behavior can be explained by the fact that D[3,2] is more influenced by the presence of smaller particles, while D[4,3] is more affected by the larger ones, which are more resistant to micronization [6,33,38]. The D[4,3] value for the control sample was nearly four times higher than the D[3,2] value, underscoring the greater proportion of larger particles in the suspension.

Significantly, the results clearly demonstrate that HPH treatment, particularly after 10 passes, effectively disrupted plant cells, facilitating the release of a substantial portion of intracellular content into the suspension.

### 3.5. Interfacial Tension and Thermal Stability of Tomato Pomace Aqueous Suspensions

**Figure 4:**
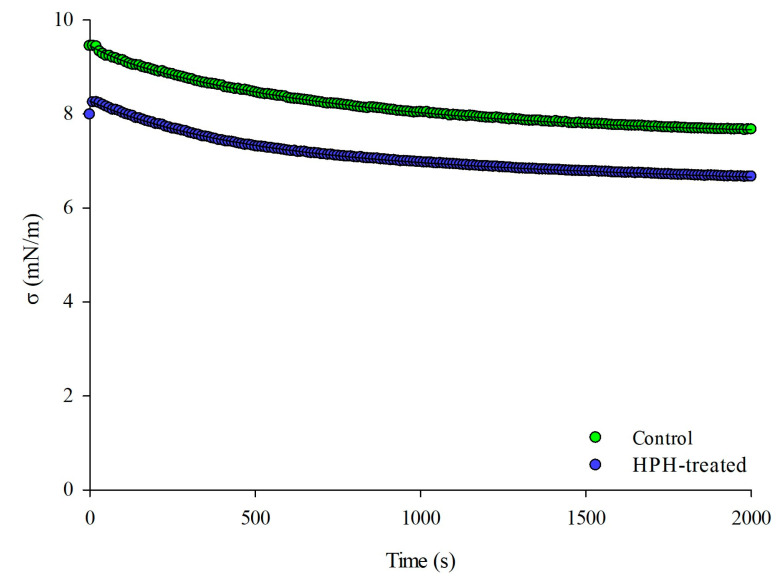
Interfacial tension, σ, of the supernatants from untreated (Control, np = 0), and HPH-treated (P = 80 MPa, T = 25 °C, np = 10) tomato pomace aqueous suspensions. The solid lines represent model fits to the experimental data.

The dynamic adsorption behavior of the suspensions was examined by monitoring changes in interfacial tension (γ) over time (0–2000 s) at 25 °C, using the pendant-drop method. Figure 4 presents the results of interfacial tension measurements at the oil-water interface for both untreated (control) and HPH-treated (P = 80 MPa, T = 25 °C, np = 10) suspensions.

The results indicate that, regardless of whether HPH treatment was applied or not, the interfacial tension of the suspensions rapidly decreased during the initial 500 s. This initial decrease is attributed to the adsorption of hydrophilic oil components, eventually reaching equilibrium [39]. Both samples exhibited a reduction in interfacial tension over time, converging toward equilibrium values (σ_∞_). These equilibrium values were estimated using the exponential decay model described by Pirozzi et al. (2023) [39], with R^2^ values ranging between 0.997 and 0.999.

Notably, the control sample exhibited a higher interfacial tension value compared to the HPH-treated sample. The asymptotic interfacial tension (σ_∞_) estimated for the HPH-treated sample (7.035 Nm/m) was significantly lower than that estimated for the control sample (8.037 Nm/m). This substantial reduction suggests that micronized tomato pomace particles have an enhanced ability to interact at the oil-water interface, highlighting the potential stabilizing effect of HPH on the suspensions.

These findings align with those reported by Juric et al. (2019) [6], who observed a significant reduction in oil-water interfacial tension for the supernatant of HPH-treated tomato peel suspension compared to the control (−15.0%). The authors suggested that the release of intracellular macromolecules, such as proteins and polysaccharides, induced by HPH treatment, could influence the interfacial tension of the aqueous phase of tomato peel suspensions, potentially leading to its reduction.

**Figure 5:**
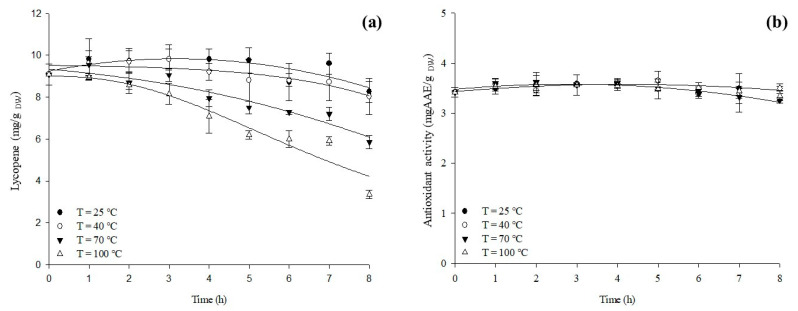
Isothermal degradation of lycopene (**a**) and antioxidant activity (**b**) in tomato pomace aqueous suspensions exposed at different temperatures (25, 40, 70, 100 °C). The solid lines represent model fits to the experimental data. The bars represent the mean ± standard deviation.

While product quality is undoubtedly crucial, ensuring the high bioactivity of active ingredients post-processing, storage, and preparation is even more significant [40]. In this context, the thermal stability kinetics of the tomato pomace suspensions, exposed to various temperatures (25, 40, 70, 100 °C) over time, were determined and presented in Figure 5. The isothermal degradation curves provide insight into how different temperatures affect lycopene content and antioxidant activity values in HPH-treated (P = 80 MPa, T = 25 °C, np = 10) suspensions.

Analyzing the thermal stability kinetics of lycopene, it becomes apparent that although a slight decrease in lycopene content is noticeable, no statistically significant differences were detected between the samples exposed to 25 °C and 40 °C compared to the initial lycopene content (t = 0 h). This phenomenon might be attributed to the complex nature of the system, encompassing multiple components. The hydrophobic character of lycopene could lead to the formation of molecular complexes, potentially enhancing its chemical stability [41]. However, clear degradation of lycopene is evident when the extracts are subjected to temperatures of 70 °C and 100 °C, resulting in a 1.5-fold and 2.7-fold reduction, respectively, after 8 h of exposure compared to the initial suspension (t = 0 h). Carotenoids like lycopene possess conjugated systems of double bonds that are susceptible to oxidation and isomerization, particularly during harsh thermal processing conditions [29].

Regarding the thermal stability kinetics of antioxidant activity (Figure 5b), it can be observed that, although not statistically significant, a decrease in antioxidant activity becomes evident as the temperature increases. This observation might be explained by the degradation of some bioactive compounds, resulting in a reduction of their antioxidant activity. However, this decline could be counterbalanced by the increased diffusion and release of other bioactive compounds from cellular walls due to the elevated temperature [42,43]. This behavior is especially prominent in the case of the total HPH-treated extract, where cell debris is present in the final suspensions. Moreover, complexation with concurrently extracted macromolecules (such as polysaccharides and proteins) to form colloidal particles might stabilize the compounds without noticeable degradation [29]. For instance, the application of HPH on orange pulp led to a more homogeneous appearance, smoother suspension, and an increased presence of water-extractable pectin, ultimately enhancing the physicochemical stability of the suspensions [44]. Among the tomato polyphenols, phenolic acids and flavonoids have the capability to strongly bind to polymers, particularly proteins or sugars, which can inhibit the autoxidation of polyphenols and contribute to high antioxidant activities [45].

### 3.6. Lycopene Bioaccessibility

Bioaccessibility in the gut, defined as the amount potentially absorbable from the lumen [46], is a critical factor in determining the potential health benefits of any compound. Lycopene, known for its poor chemical stability and low oral bioavailability, presents limitations in realizing its health benefits. However, its fate after consumption is often overlooked [19], making in vitro release studies essential when developing and validating new food products.

**Figure 6:**
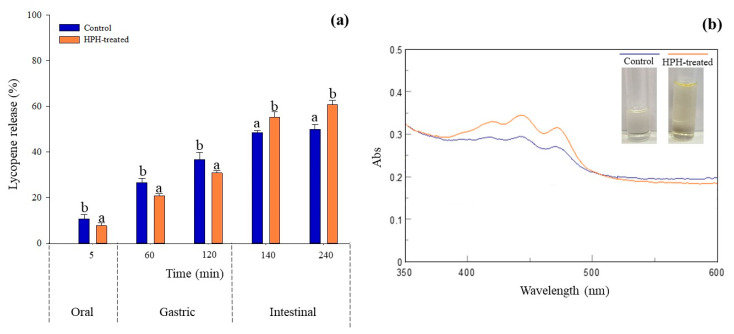
UV-vis spectra of lycopene released from untreated (control) and HPH-treated tomato pomace suspensions in the intestinal phase during in vitro digestion, (**a**). Release (%) of lycopene content during simulated digestion from the untreated and HPH-treated suspensions, (**b**). Bars related to the samples at the same digestion time bearing different lowercase letters indicate significant differences (*p* < 0.05).

In this context, the bioaccessibility of lycopene in both untreated and HPH-treated (P = 80 MPa, T = 25 °C, np = 10) tomato pomace suspensions was assessed using an in vitro digestion model (Figure 6).

The results indicate that the bioaccessibility of lycopene during the intestinal phase ranged from 50.0% to 60.9%, with both samples displaying similar digestion profiles. On average, lycopene release was approximately 9.28% during the oral phase, 33.8% during the gastric phase, and 55.4% during the intestinal phase.

While variability is attributed to factors such as raw materials, applied treatments, and methodologies, the bioaccessibility values observed in this study are consistent with findings from previous research on lycopene bioaccessibility in various tomato products, such as tomato juice [19,47], canned tomatoes [48], tomato emulsions [49], and tomato paste [50], ranging from 2.11% to 46%.

During the intestinal phase, carotenoids like lycopene are typically incorporated into micelles formed by bile acids, free fatty acids, glycerides, and phospholipids [49]. These micelles contain the bioaccessible lycopene. Notably, in line with the lycopene recovery yields (Table 1), the UV-vis spectra of lycopene released in the simulated intestinal fluid (SIF), shown in Figure 6b, confirmed a higher lycopene content in the micellar phase of the digested HPH-treated tomato pomace suspension compared to the control.

The sustained increase in lycopene during the intestinal phase suggests that pancreatin and pH conditions in this stage promote the release of bioactive compounds by facilitating the degradation of macromolecules into sugars and small peptides [21]. However, the control sample exhibited a rapid release of lycopene during the oral and gastric phases, followed by a slower release in the intestinal phase (Figure 6a). In contrast, the HPH-treated sample displayed a more controlled release of lycopene throughout the simulated fluids, resulting in higher bioaccessibility (+22%) compared to the control.

These differences between the control and HPH-treated samples can be attributed to structural and bonding pattern changes induced by HPH treatment, influencing the type and strength of interactions and thus affecting lycopene release during gastrointestinal digestion. High-pressure treatments, like HPH, can break weak bonds in macromolecules, potentially inducing hydrophobic interactions that lead to the exchange or formation of new bonds [51,52].

Moreover, the decrease in viscosity induced by HPH may prevent lycopene from interacting with bile salts, promoting micelle formation and ultimately enhancing lycopene bioaccessibility under high-pressure conditions. Similar findings were reported by other researchers in tomato purees and pulp [13,17,20]. For example, Zhang et al. (2019) [19] showed that the increase in lycopene bioaccessibility upon the HPH treatment was attributed to the isomerization of all-trans lycopene and disruption and rebuilding of the fiber and hydrocolloidal structure of tomato juice. The decrease in viscosity induced by the HPH was connected to preventing the interaction of lycopene and bile salt, by promoting micelle formation and improving the lycopene bioaccessibility at high pressure [19].

Likewise, Knokaert et al., 2012 [20] and Colle et al., 2010 [13] found that HPH treatment enhanced the bioaccessible lycopene in tomato purees and pulp, respectively.

However, future studies should aim to clarify the role of other components present in tomato pomace suspensions, including proteins, fibers, and colloids, in influencing the bioaccessibility of lycopene.

## 4. Conclusions

Emerging technologies like High-Pressure Homogenization (HPH) offer promising opportunities to enhance the quality and nutritional value of tomato products, particularly by utilizing industrial by-products. In this study, the effects of HPH processing parameters (temperature, pressure, and number of passes) on several key response variables (Total Phenolic Content—TPC, Ferric Reducing Antioxidant Power—FRAP, and lycopene content) were investigated using response surface methodology. The results revealed significant effects of HPH treatment on tomato pomace suspensions.

Specifically, applying HPH at the optimal processing conditions (P = 80 MPa, T = 25 °C, np = 10) resulted in several improvements compared to the control. HPH treatment led to a 31% increase in TPC, a 25% increase in FRAP values, and a substantial 42% increase in lycopene content. This indicates that HPH can effectively release and recover valuable bioactive compounds from tomato pomace. Moreover, the treatment enhanced the consistency and homogeneity of the suspensions, making them more suitable for use as a food additive. HPH treatment significantly reduced particle size, leading to a seven-fold reduction. This not only improves the texture and appearance of the suspensions but also enhances their stability. Additionally, the interfacial tension of the suspensions decreased by 15%, which can be advantageous for stabilizing the suspensions, potentially making them suitable for various food applications. Finally, HPH treatment improved the bioaccessibility of lycopene by 22%, with a more controlled release of lycopene during simulated gastrointestinal digestion compared to the control. This suggests that HPH can potentially enhance the bioavailability of lycopene, making it more beneficial when consumed.

The study’s findings hold promise for the development of homogeneous and smooth tomato pomace suspensions as natural food additives. This could be particularly valuable for food products where maintaining sensory characteristics is essential. Additionally, this approach has the potential to drive technological advancements in HPH, opening up new possibilities for large-scale production of tomato products with improved physicochemical properties and health benefits, all starting from cost-effective raw materials like tomato pomace. This could have significant implications for both the food industry and sustainable resource utilization.

## Figures and Tables

**Table 1:** Actual values of the three independent variables investigated and three response variables (TPC, FRAP, and lycopene) in untreated and HPH-treated suspensions.

Run	Independent Variables			Dependent Variables		
	T (°C)	P (MPa)	np (–)	TPC (mg GAE/g _DW_)	FRAP (mg AAE/g _DW_)	Lycopene Content (mg/g _DW_)
Untreated	/	/	0	3.30 ± 0.40	3.63 ± 0.05	7.20 ± 0.10
1	5	80	2	2.67 ± 0.30	3.71 ± 0.00	8.55 ± 0.10
2	25	80	2	3.53 ± 0.01	4.47 ± 0.10	8.65 ± 0.10
3	50	80	2	3.44 ± 0.10	3.75 ± 0.02	10.10 ± 0.20
4	5	100	2	2.62 ± 0.22	3.04 ± 0.01	9.09 ± 0.10
5	25	100	2	2.71 ± 0.01	3.18 ± 0.01	9.56 ± 0.10
6	50	100	2	2.84 ± 0.05	3.49 ± 0.20	7.61 ± 0.15
7	5	80	5	3.26 ± 0.11	4.01 ± 0.10	9.77 ± 0.20
8	25	80	5	3.31 ± 0.01	4.76 ± 0.15	9.33 ± 0.02
9	50	80	5	4.07 ± 0.20	4.73 ± 0.50	8.66 ± 0.01
10	5	100	5	2.95 ± 0.10	3.08 ± 0.20	8.93 ± 0.35
11	25	100	5	2.97 ± 0.02	2.93 ± 0.30	9.55 ± 0.20
12	50	100	5	2.93 ± 0.07	3.65 ± 0.20	6.25 ± 0.01
13	5	80	10	3.69 ± 0.10	3.79 ± 0.20	9.90 ± 0.10
14	25	80	10	4.33 ± 0.08	4.52 ± 0.04	9.55 ± 0.10
15	50	80	10	3.77 ± 0.05	4.75 ± 0.30	8.32 ± 0.10
16	5	100	10	3.12 ± 0.05	3.35 ± 0.15	8.28 ± 0.20
17	25	100	10	3.21 ± 0.05	3.20 ± 0.10	9.47 ± 0.25
18	50	100	10	3.34 ± 0.05	3.95 ± 0.02	5.90 ± 0.30
19	5	80	15	3.85 ± 0.05	4.17 ± 0.20	10.03 ± 0.21
20	25	80	15	3.54 ± 0.05	4.57 ± 0.10	10.20 ± 0.10
21	50	80	15	4.05 ± 0.05	4.73 ± 0.30	8.15 ± 0.10
22	5	100	15	3.41 ± 0.05	3.06 ± 0.25	7.13 ± 0.15
23	25	100	15	3.46 ± 0.15	3.37 ± 0.30	7.48 ± 0.20
24	50	100	15	3.67 ± 0.10	4.03 ± 0.20	5.81 ± 0.10

**Table 2:** Proximate composition and visual pesticide detection of tomato pomace.

Moisture (g/100 g _FW_)	Ash (g/100 g _DW_)	Protein (g/100 g _DW_)	Fat (g/100 g _DW_)	Carbohydrates (g/100 g _DW_)	Total Fibre (g/100 g _DW_)	Pesticides
80.7 ± 0.8	4.9 ± 0.3	14.7 ± 0.2	1.3 ± 0.1	5.8 ± 0.5	73.3 ± 0.9	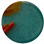 (absent) *

* The image illustrates the visual outcome of the pesticide test using the Agri-Screen Ticket Pesticide Detection Kit. A white ticket, without any color change, indicates a positive response (presence of pesticides), whereas a color shift of the ticket to clear blue signifies a negative response (absence of pesticides).

**Table 3:** Analysis of variance (ANOVA) of the two-factor interaction (2FI) model describing the influence of the HPH-treatment parameters on the TPC, FRAP, and lycopene content of tomato pomace suspensions.

Coefficients	TPC (mgGAE/g _DW_)		FRAP (mgAAE/g _DW_)		Lycopene (mg/g _DW_)	
β_0_	4.7842		7.90789		6.12308	
β_1_ (T)	0.039307	*	−0.002674	***	0.117652	**
β_2_ (P)	−0.023589	***	−0.049396	***	0.036754	*
β_3_ (np)	−0.011305	***	0.021533	*	0.724928	**
β_12_ (T · P)	−0.000318	ns	0.000103	ns	−0.001457	*
β_13_ (T · np)	−0.000457	ns	0.000813	ns	−0.002269	*
β_23_ (P · np)	0.000830	ns	−0.000187	ns	−0.008056	*
*p*-value of the model	0.0003	***	<0.0001	***	0.0009	***
R^2^	0.744		0.843		0.703	
RMSE	0.041		0.019		0.039	

ns: not significant for *p* > 0.05. * significant for *p* ≤ 0.05; ** significant for *p* ≤ 0.01; *** significant for *p* ≤ 0.001. RMSE, Root Mean Square Error.

**Table 4:** Color parameters (L*, a*, b*), color differences (ΔΕ*) among untreated and HPH-treated tomato pomace suspensions, hue angle (h*_ab_), and chroma (C*_ab_) of suspensions as a function of HPH passes, evaluated at P = 80 MPa and T in the range 5–50 °C.

T = 5 °C						
HPH passes	L*	a*	b*	ΔE*	h*_ab_	C*_ab_
0	32.33 ± 0.02 ^a^	14.86 ± 0.03 ^a^	22.82 ± 0.03 ^a^	–	56.93 ± 0.80 ^a^	27.23 ± 0.01 ^a^
2	36.55 ± 0.05 ^e^	15.05 ± 0.10 ^b^	23.64 ± 0.04 ^b^	4.30 ± 0.06 ^d^	57.52 ± 0.60 ^a^	28.02 ± 0.04 ^b^
5	33.64 ± 0.05 ^b^	15.77 ± 0.22 ^d^	24.79 ± 0.04 ^c^	2.53 ± 0.06 ^ab^	57.53 ± 0.50 ^a^	29.38 ± 0.03 ^d^
10	33.77 ± 0.01 ^c^	15.26 ± 0.05 ^c^	24.78 ± 0.04 ^c^	2.46 ± 0.05 ^a^	58.37 ± 0.50 ^b^	29.10 ± 0.04 ^c^
15	34.07 ± 0.01 ^d^	14.90 ± 0.04 ^a^	26.63 ± 0.04 ^d^	4.19 ± 0.01 ^c^	60.77 ± 0.50 ^bc^	30.51 ± 0.05 ^e^
T = 25 °C						
HPH passes	L*	a*	b*	ΔE*	h*_ab_	C*_ab_
0	32.45 ± 0.01 ^a^	14.86 ± 0.04 ^a^	24.09 ± 0.02 ^b^	–	58.33 ± 0.70 ^b^	28.30 ± 0.01 ^b^
2	35.96 ± 0.04 ^d^	14.82 ± 0.05 ^a^	23.98 ± 0.04 ^a^	3.52 ± 0.04 ^c^	58.28 ± 0.50 ^b^	28.19 ± 0.02 ^a^
5	34.08 ± 0.00 ^b^	15.89 ± 0.02 ^b^	24.12 ± 0.02 ^b^	1.93 ± 0.05 ^a^	56.62 ± 0.50 ^a^	28.88 ± 0.03 ^c^
10	34.8 ± 0.01 ^c^	16.77 ± 0.02 ^c^	25.3 ± 0.02 ^c^	3.26 ± 0.01 ^b^	56.46 ± 0.40 ^a^	30.35 ± 0.02 ^d^
15	34.79 ± 0.01 ^c^	17.69 ± 0.03 ^d^	26.44 ± 0.05 ^d^	4.36 ± 0.04 ^d^	56.21 ± 0.70 ^a^	31.81 ± 0.04 ^e^
T = 50 °C						
HPH passes	L*	a*	b*	ΔE*	h*_ab_	C*_ab_
0	33.02 ±0.02 ^a^	14.86 ±0.06 ^d^	23.68 ±0.04 ^a^	–	57.89± 0.76 ^a^	27.96± 0.06 ^a^
2	37.58 ±0.06 ^e^	12.82 ±0.03 ^c^	24.73 ±0.06 ^b^	5.11± 0.04 ^a^	62.60± 0.80 ^b^	27.86± 0.06 ^a^
5	36.22 ±0.01 ^c^	11.1 ±0.03 ^b^	25.93 ±0.01 ^c^	5.43± 0.02 ^b^	66.82± 0.60 ^c^	28.21± 0.02 ^b^
10	35.27 ±0.01 ^b^	10.57 ±0.03 ^a^	26.89 ±0.03 ^d^	5.81± 0.01 ^c^	68.54± 0.55 ^d^	28.89± 0.04 ^c^
15	36.32 ±0.01 ^d^	10.54 ±0.03 ^a^	29.16 ±0.04 ^e^	7.72± 0.02 ^d^	70.13± 0.70 ^e^	31.01± 0.04 ^d^

Values with different lowercase letters within the same column and for each operating temperature are significantly different (*p* ≤ 0.05).

## Data Availability

The data presented in this study are available in the article and in the associated Supplementary Material. Raw data are available on request from the corresponding author.

## Supplementary Materials

The following supporting information can be downloaded at: https://www.mdpi.com/article/10.3390/antiox12101855/s1, Figure S1: Scatter plot of predicted values vs actual values from experimental design for TPC (**a**), FRAP values (**b**), and lycopene content (**c**). Black points represent independent experimental design points obtained at the optimized HPH processing conditions for model validation; Figure S2: HPLC-PDA chromatograms of untreated (control, blue line) and HPH-treated (P = 80 MPa, T = 25 °C, np = 10, red line) suspensions from tomato pomace. Peak identification: lycopene. Ref. [53] is cited in Supplementary Materials file.
