# Supplementary material for: High-Pressure Homogenization for Enhanced Bioactive Recovery from Tomato Processing By-Products and Improved Lycopene Bioaccessibility during In Vitro Digestion
Source: Antioxidants (Basel). 2023 Oct 13;12(10):1855. doi: 10.3390/antiox12101855 (PMC10603967; doi:10.3390/antiox12101855)
Supplement: Supplementary file 1 [file antioxidants-12-01855-s001.zip › antioxidants-2657949-supplementary.pdf]

antioxidants

Supplementary materials

High-Pressure Homogenization for Enhanced Bioactive Recovery from Tomato Processing By-Products and Improved Lycopene Bioaccessibility during In Vitro Digestion

Serena Carpentieri [1], Giovanna Ferrari [1,2] and Francesco Donsì [1,3,*]

[1] Department of Industrial Engineering, University of Salerno, Via Giovanni Paolo II, 132, 84084 Fisciano, Italy; scarpentieri@unisa.it (S.C.); gferrari@unisa.it (G.F.)
[2] ProdAl Scarl, c/o University of Salerno, Via Giovanni Paolo II, 132, 84084 Fisciano, Italy
[3] NBFC (National Biodiversity Future Center), 90133 Palermo, Italy
* Correspondence: fdonsi@unisa.it; Tel.: +39-089-964135

Materials and Methods

S1. HPLC-PDA analysis

A Waters 1525 Separation Module coupled to a photodiode array detector Waters 2996 (Waters Corporation, Milford, MA, USA) was used for the high-performance liquid chromatography–photodiode array detection (HPLC–PDA) analysis for the identification of lycopene in the suspensions of untreated (control) and HPH-treated tomato pomace, according to the method reported by Carpentieri et al. (2023). The suspensions were first centrifugated and filtered with a 0.45 μm filter, and injected into the column (injection volume 5 μL). The signal for the quantification of lycopene was reported at the wave- length of maximum absorbance λ = 472 nm. The retention times of the identified com- pounds were compared with those of lycopene commercial standard.

Results and discussion

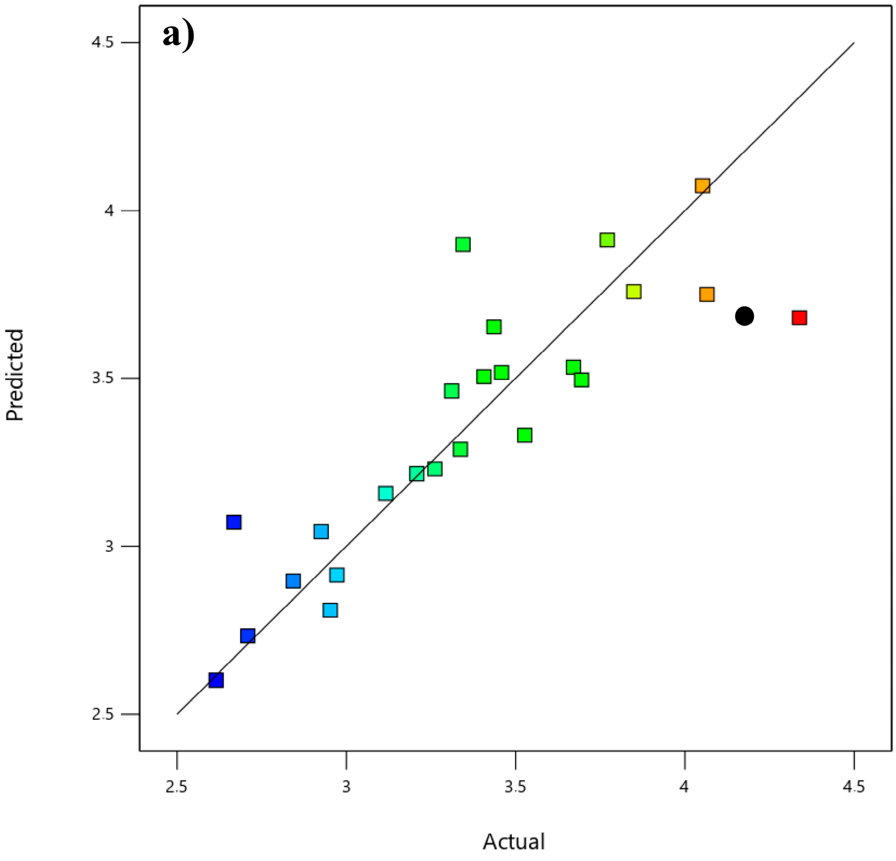

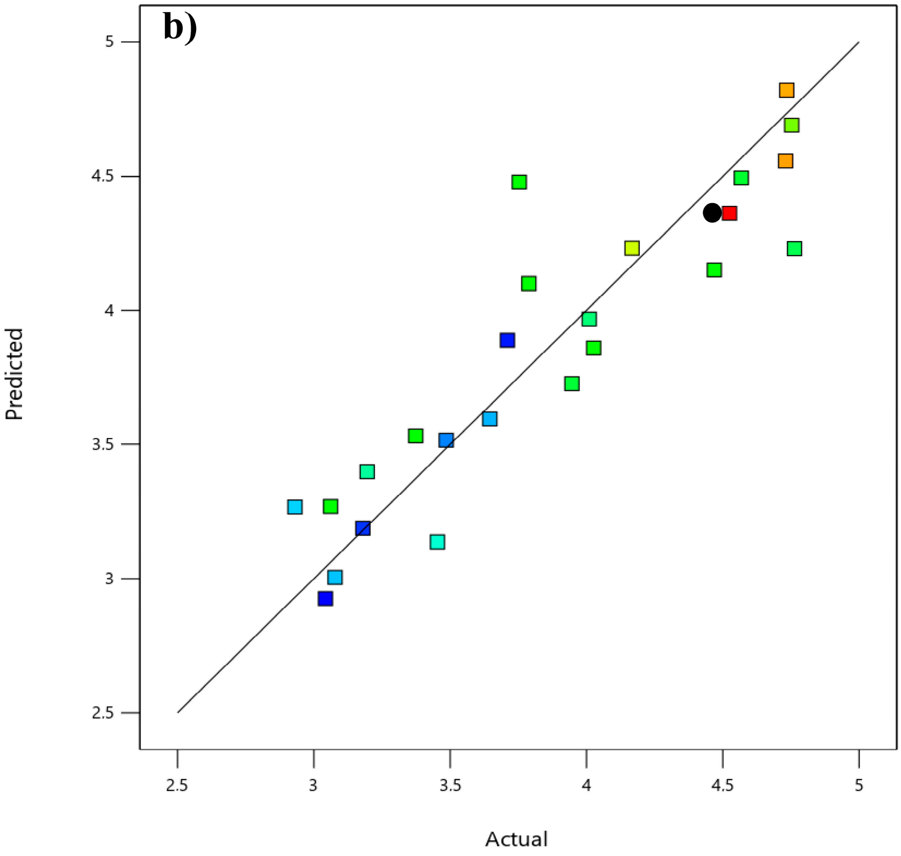

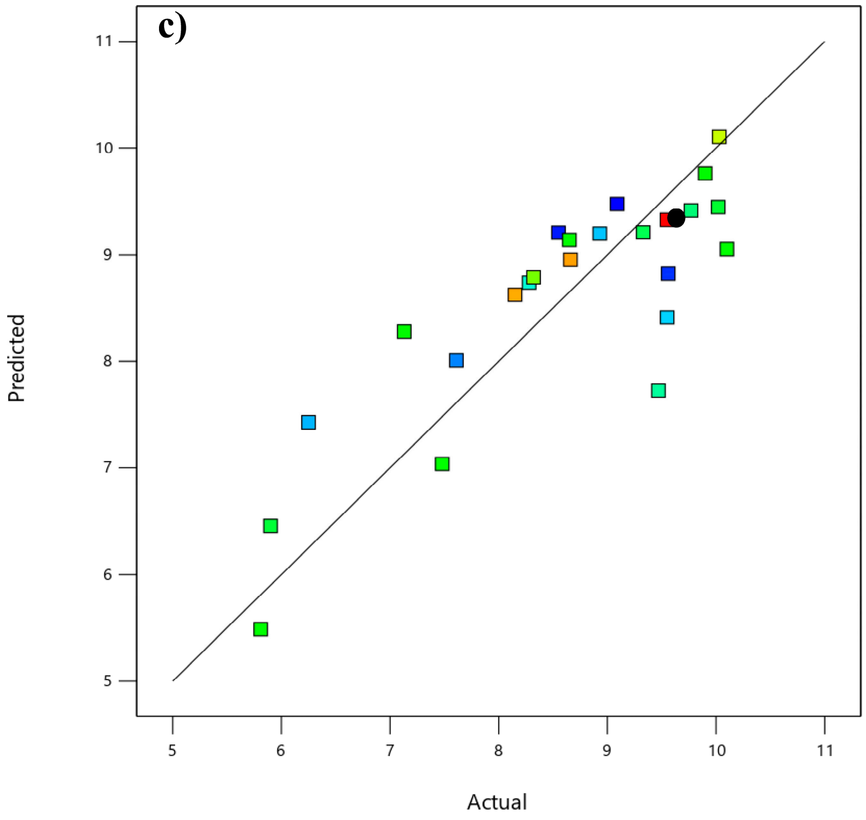

Figure S1. Scatter plot of predicted values vs actual values from experimental design for TPC (a), FRAP values (b), and lycopene content (c). Black points represent independent experimental design points obtained at the optimized HPH processing conditions for model validation.

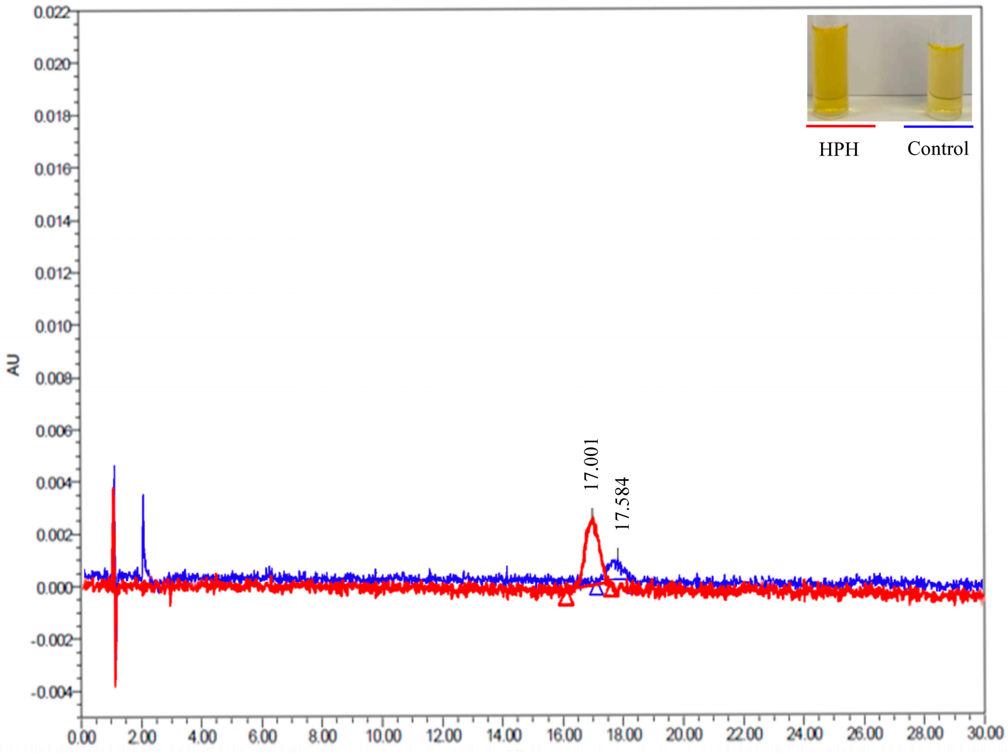

Figure S2. HPLC-PDA chromatograms of untreated (control, blue line) and HPH-treated (P = 80 MPa, T = 25 °C, np = 10, red line) suspensions from tomato pomace. Peak identification: lycopene.

References

Carpentieri, S., Ferrari, G., Pataro, G. Pulsed Electric Fields-Assisted Extraction of Valuable Compounds from Red Grape Pomace: Process Optimization Using Response Surface Methodology. *Front. Nutr.* **2023**, *10*, 1158019. https://doi.org/10.3389/fnut.2023.1158019.
